# Large-scale public data reuse to model immunotherapy response and resistance

**DOI:** 10.1186/s13073-020-0721-z

**Published:** 2020-02-26

**Authors:** Jingxin Fu, Karen Li, Wubing Zhang, Changxin Wan, Jing Zhang, Peng Jiang, X. Shirley Liu

**Affiliations:** 1 Clinical Translational Research Center, Shanghai Pulmonary Hospital, School of Life Science and Technology, Tongji University, Shanghai, 200433 China; 2 Department of Data Sciences, Dana Farber Cancer Institute, Harvard T.H. Chan School of Public Health, Boston, MA 02215 USA; 3 Tongji Hospital, School of life Science and Technology, Tongji University, Shanghai, 200065 People’s Republic of China; 4 The Winsor School, Boston, MA 02215 USA; 5 Present Address: Cancer Data Science Laboratory, National Cancer Institute, National Institutes of Health, Bethesda, MD 20892 USA

**Keywords:** Immunotherapy, Immune evasion, Data integration, Web platform

## Abstract

Despite growing numbers of immune checkpoint blockade (ICB) trials with available omics data, it remains challenging to evaluate the robustness of ICB response and immune evasion mechanisms comprehensively. To address these challenges, we integrated large-scale omics data and biomarkers on published ICB trials, non-immunotherapy tumor profiles, and CRISPR screens on a web platform TIDE (http://tide.dfci.harvard.edu). We processed the omics data for over 33K samples in 188 tumor cohorts from public databases, 998 tumors from 12 ICB clinical studies, and eight CRISPR screens that identified gene modulators of the anticancer immune response. Integrating these data on the TIDE web platform with three interactive analysis modules, we demonstrate the utility of public data reuse in hypothesis generation, biomarker optimization, and patient stratification.

## Background

Despite growing numbers of published immune checkpoint blockade (ICB) trials in different cancer types with available omics data and clinical outcomes, ICB response prediction remains an open question. Many published ICB response biomarkers had been trained and tested on limited cohorts and showed variable performance in different cohorts. Moreover, with the limited data size in each clinical study, it is challenging to comprehensively evaluate the complexity of ICB response and immune evasion mechanisms. To address these challenges, we present a data-driven approach integrating large-scale omics data and biomarkers on published ICB trials, non-immunotherapy tumor profiles, and CRISPR screens on a web platform TIDE (http://tide.dfci.harvard.edu).

Previously, we developed TIDE as a transcriptome biomarker of ICB response by modeling tumor immune dysfunction and exclusion [1]. The statistical model of TIDE was trained on clinical tumor profiles without ICB treatments since the immune evasion mechanisms in treatment-naïve tumors are also likely to influence patient response to immunotherapies. The TIDE model has been applied to evaluate T cell dysfunction and exclusion signatures across over 33K samples in 188 tumor cohorts from well-curated databases, including TCGA [2], METABRIC [3], and PRECOG [4], as well as our in-house collections. In the current work, we significantly expanded the scope of our previous work by incorporating many new datasets and function modules (Additional file 1: Table S1).

## Construction and content

**Fig. 1:**
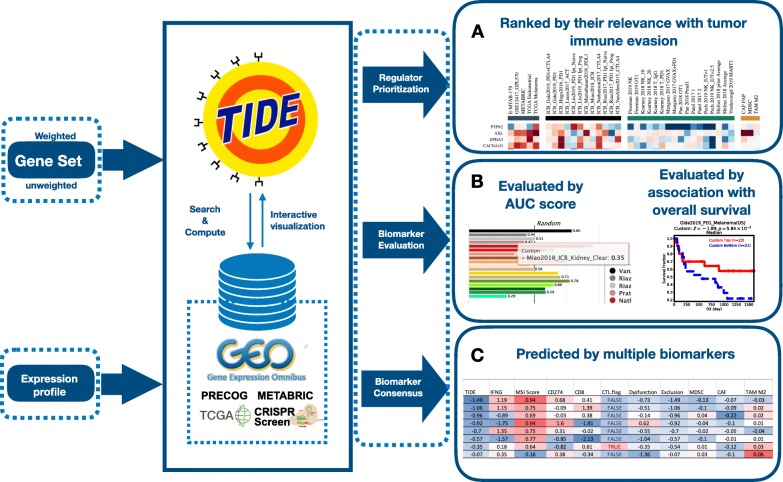
TIDE web platform architecture. The TIDE web platform aims to facilitate the hypothesis generation, biomarker optimization, and patient stratification in immune-oncology research through a public data reuse approach. The platform functions are based on the integration of large-scale omics data and biomarkers on published ICB trials, non-immunotherapy tumor profiles, and CRISPR screens. The web platform takes gene set or expression profiles as input and provides three interactive modules. **A** Gene prioritization for a user-input gene set. Every gene is ranked by their clinical relevance and CRISPR screen phenotype, including four types of metrics: 1, the association between gene expression and T cell dysfunction across cohorts, computed as the z-score in the Cox Proportional Hazard (PH) regression model; 2, the association between gene expression and ICB response across tumors, computed as the *z*-score in the Cox-PH regression; 3, the log-fold change in CRISPR screens probing the effect of gene knockout on lymphocyte-mediated tumor killing; 4, the gene expression in cell types driving T cell exclusion in tumors. Data cohorts are grouped by their metric types on the heatmap (columns). Genes (rows) can be interactively reordered by the gene values either on a single data set or any metric type groups. **B** Biomarker evaluation for a custom biomarker gene set. The predictive power of biomarkers in the public immunotherapy cohorts is quantified by two criteria, the area under the receiver operating characteristic curve (AUC) and the z-score in the Cox-PH regression. We visualize biomarkers’ AUC by bar plots (left panel) and Cox-PH z-scores by Kaplan-Meier curve (right panel). **C** Biomarker consensus to predict ICB response from gene expression profile. Every input transcriptomic profile is evaluated by TIDE, microsatellite instability (MSI) signature, interferon-gamma (IFNG) signature, and other biomarkers reported in the literature

We processed the omics data for 998 tumors from 12 published ICB clinical studies (listed in Additional file 2: Table S2), and eight published CRISPR screens that identified genes modulating lymphocyte-mediated cancer killing and immunotherapy response [5–8]. The clinical study data from ICB naïve cohorts includes 33K samples in 188 tumor cohorts from well-curated databases, including TCGA [2], METABRIC [3], and PRECOG [4]. We integrated these data on the TIDE web platform using the MySQL database. The web platform is based on the Django 3.0 framework. We provided three interactive modules for hypothesis generation, biomarker optimization, and patient stratification (Fig. 1).

## Utility and discussion

### Gene set prioritization module

The first module of the TIDE web platform can help cancer biologists prioritize genes in their input gene set for mechanistic follow-up experiments (Fig. 1A). Typically, a genomic experiment, often conducted on model systems in limited sample size, will yield tens to hundreds of gene hits. The large-scale omics data and clinical cohorts collected in TIDE enable cancer biologists to focus on genes with the highest clinical relevance and consistent behavior in other similar experiments. Generally, for any gene sets, a cancer biologist can utilize this module to evaluate each gene for its expression associations with ICB response outcome, T cell dysfunction levels, T cell exclusion levels, and phenotypes in genetic screens in diverse cohorts. To probe a candidate gene further, the user can also use a single gene as query to evaluate how the expression, copy number, somatic mutation, and DNA methylation levels of this gene influence clinical outcome in all collected datasets. Therefore, the prioritization module, integrating many independent cohorts, can help identify genes with improved robustness and clinical relevance.

**Fig. 2:**
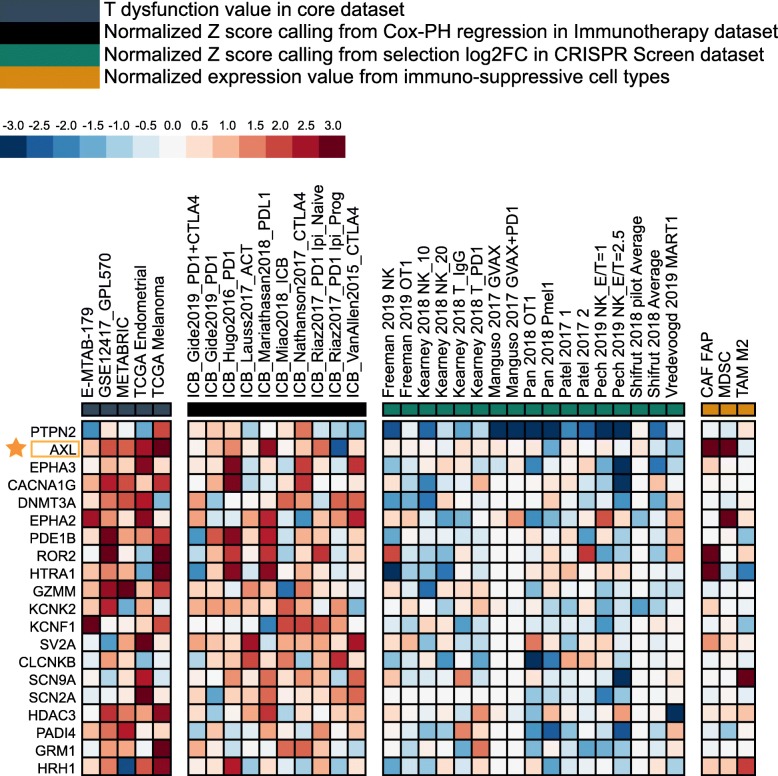
Prioritization of genes with approved drugs. A total of 696 genes with launched drugs were collected from the OASIS database [9] (Additional file 5: Table S4). Among the gene set, top 20 hits were presented. Genes (row) are ranked by their weighted average value across four immunosuppressive indices (columns), including T cell dysfunction score, T cell exclusion score, association with ICB survival outcome, and log-fold change (logFC) in CRISPR screens. The T dysfunction score shows how a gene interacts with cytotoxic T cells to influence patient survival outcome, and the T cell exclusion score assesses the gene expression levels in immunosuppressive cell types that drive T cell exclusion. The association score of (*z*-score in the Cox-PH regression) ICB survival outcome evaluates genes whose activities are correlated with ICB benefit. The normalized logFC in CRISPR screens help identify regulators whose knockout can mediate the efficacy of lymphocyte-mediated tumor killing in cancer models

To demonstrate an example of usage of the regulator prioritization module, we queried 696 druggable genes annotated by the OASIS database [9], to find potential therapeutic targets in synergy with ICB (Fig. 2). For example, *AXL*, a Tyro3/Axl/Mer family receptor tyrosine kinase, is among the top targets ranked by this module to render the tumor microenvironment resistant to ICB. High *AXL* expression is associated with T cell dysfunction phenotypes in all datasets enumerated (Fig. 2 left panel). Meanwhile, high expression of *AXL* is also associated with worse ICB outcome in bladder cancer and treatment-naïve melanoma treated with ICB (Fig. 2 second to left panel). Among the cell types promoting T cell exclusion, both myeloid-derived suppressor cell and cancer-associated fibroblast have very high *AXL* expression level (Fig. 2 right panel). Indeed, in a recent clinical trial NCT03184571, the combination of AXL inhibitor and anti-PD1 has shown promising efficacy among AXL-positive non-small cell lung cancer patients [10]. Hence, this module can prioritize genes with the best potential for developing combination immunotherapies.

### Biomarker evaluation module

The second module allows translational scientists to evaluate the accuracy of their biomarkers on many ICB cohorts in comparison with other published biomarkers (Fig. 1B). We implemented eight published ICB response biomarkers and applied them to our collection of published ICB trial samples. For a user-defined custom biomarker, which can be a gene set or weighted gene score vector, this module calculates the biomarker expression level in all ICB cohorts. The module displays the comparison between the custom biomarker and other published biomarkers based on their predictive power of response outcome and overall survival.

**Fig. 3:**
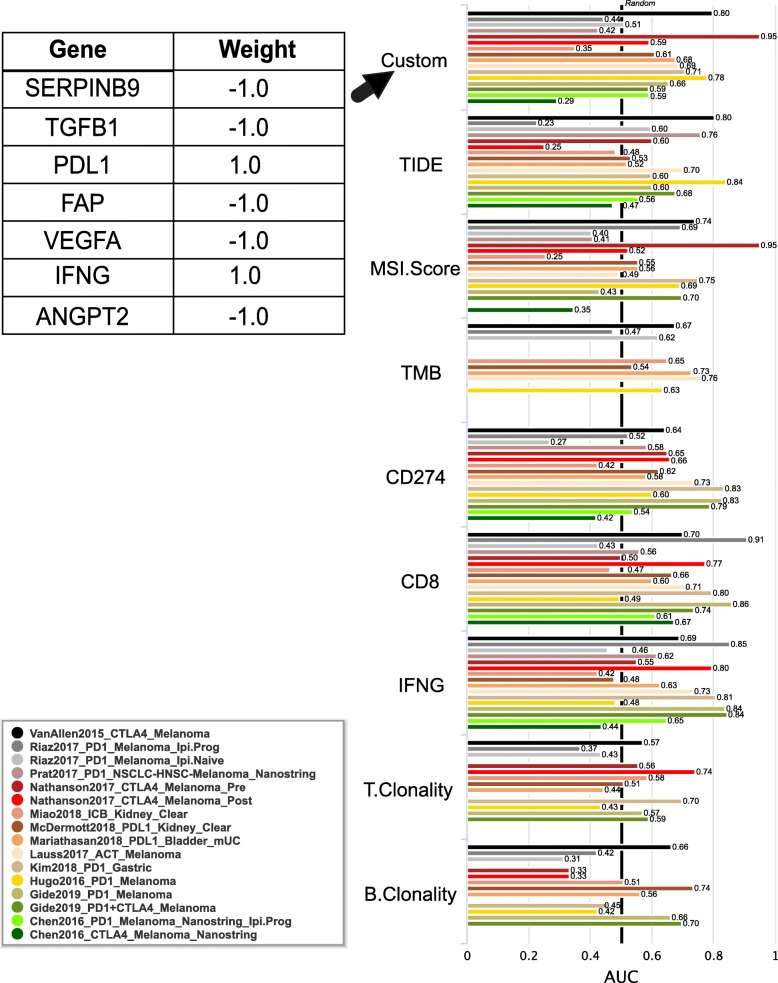
Comparison of biomarkers. The test biomarker is composed of genes with consistent evidence on cancer immune evasion (Additional file 3: Table S3). The area under receiver operating characteristic curve (AUC) is applied to evaluate the prediction performance of the test biomarker on the ICB response status

**Fig. 4:**
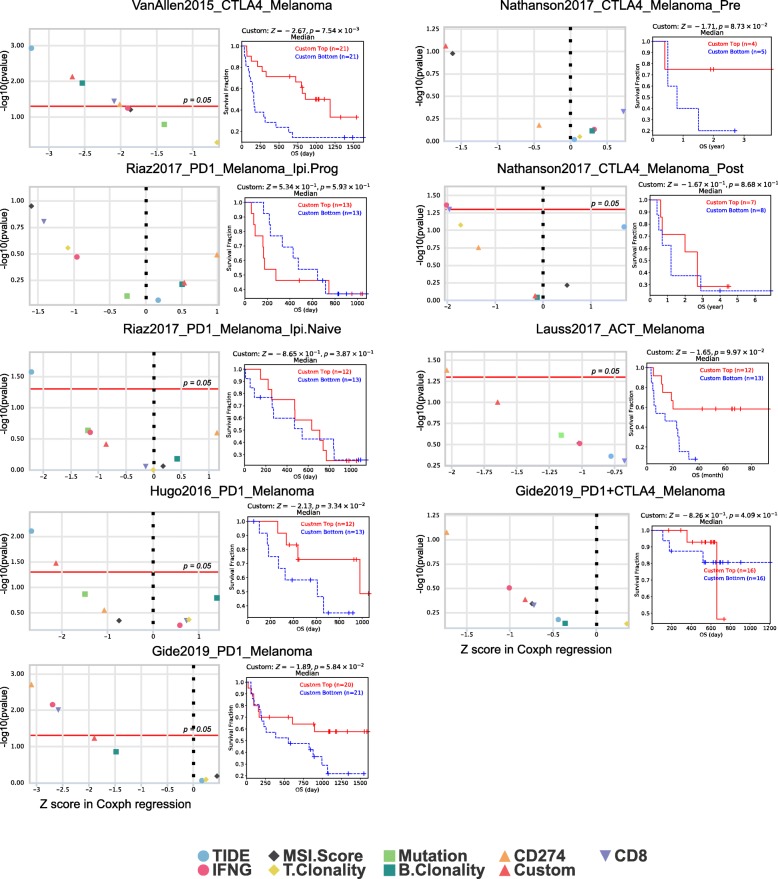
Comparison of biomarkers based on their association with overall survival. The right panel shows the association of the custom biomarker (Additional file 3: Table S3) with patients’ overall survival through Kaplan-Meier curves. In the left panel, the *x*-axis shows the *z*-score on Cox-PH regression and the *y*-axis indicates its significance level (two-sided Wald test)

To demonstrate an example usage of the biomarker evaluation module, we tested one biomarker containing seven genes with previously reported association with tumor immune evasion (Additional file 3: Table S3). These genes were weighted by their reported direction of mediating anticancer immune response. This example biomarker gave an area under the receiver operating characteristic curve (AUC) greater than 0.5 in 12 out of the 16 ICB sub-cohorts (Fig. 3), suggesting it to be a robust predictive biomarker. This signature also achieved significant associations with prolonging survival in two sub-cohorts (Fig. 4, two-sided Cox-PH *p* value < 0.05). In contrast, several recently published biomarkers trained on limited clinical cohorts have shown significant performance variations in other cohorts (Additional file 4: Figure S1), underscoring the importance of cross-cohort evaluation of biomarker robustness using all available cohorts.

### Biomarker consensus module

The third module of biomarker consensus aids oncologists in predicting whether a patient will respond to ICB therapy based on multiple biomarkers (Fig. 1C). Based on tumor pre-treatment expression profiles, oncologists could use this TIDE module and multiple published transcriptomic biomarkers (Additional file 4: Supplementary Methods) to predict patient response and potentially make informed treatment decisions. Notably, in the second and third TIDE modules, we only focused on evaluating transcriptomic biomarkers but not mutation biomarkers due to the following reasons. The results of tumor mutation analyses might be influenced by different experimental platforms (whole genome versus custom panel), sample types (FFPE versus fresh frozen), and computational mutation callers. Although tumor mutation burden (TMB) seems to be a consistent ICB response biomarker, the computation of TMB across different cohorts and platforms is still an open question.

To demonstrate an example usage of the biomarker consensus module, we upload the pre-treatment expression matrix of a melanoma cohort [11] treated with anti-PD1 therapy (Table 1). Patients with favorable predictions from multiple biomarkers are highly likely to be responders. For example, patient 2 tumor has a negative TIDE score, indicating a lack of tumor immune evasion phenotypes. In addition, patient 2 tumor has positive scores of interferon-gamma (IFNG) signature, macro-satellite instability (MSI), and PDL1 (CD274) levels, all of which are positive biomarkers of ICB response. With the support from multiple markers, an oncologist could be more confident that patient 2 will respond to anti-PD1, and indeed patient 2 is a responder in the original study [11]. In contrast, this module also reported some patients who are unlikely to benefit from ICB (Table 1). For example, patient 10 tumor has high TIDE score and low IFNG, MSI, and PDL1 levels. Based on the predictions from multiple biomarkers, an oncologist might predict patient 10 as a non-responder and select an alternative therapy, and indeed patient 10 failed to benefit from anti-PD-1 [11]. TIDE also showed that patient 10 tumor has a significant enrichment of T cell exclusion signature due to high infiltration of myeloid-derived suppressor cell (MDSC) and cancer-associated fibroblast (CAF). Therefore, elimination of MDSC and CAF might be needed for patient 10 to respond to ICB. In summary, by presenting the predictions from multiple biomarkers in one integrated platform, the biomarker consensus module can potentially inform oncologists on treatment decisions.

**Table 1:**
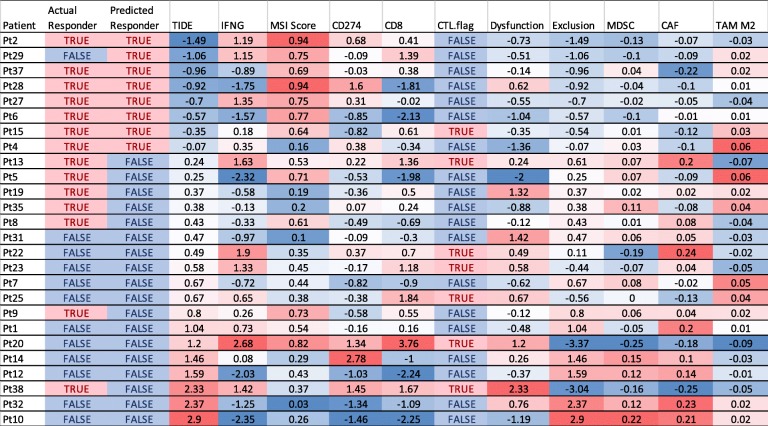
Response prediction output from the biomarker consensus module. The expression profile uploaded comes from a previous study of anti-PD1 response in melanoma [11] (“example 1” on the TIDE website). We ranked rows by ascending order of TIDE score. *Actual Responder* the actual clinical outcome in the study, *Predicted Responder* predictions by the threshold of the TIDE score set by a user (default is 0), *TIDE* TIDE prediction score [1], *IFNG* average expression of interferon-gamma response signature, *MSI Score* microsatellite instability score predicted through gene expression (Additional file 4: Supplementary Methods), *CD274* gene expression value of PD-L1, *CD8* gene expression average of CD8A and CD8B, *CTL.flag* flag indicator for whether the gene expression values are all positive for five cytotoxic T lymphocyte markers, including CD8A, CD8B, GZMA, GZMB, and PRF1, *Dysfunction*, *Exclusion*, *MDSC*, *CAF*, *TAM M2* enrichment scores based on the gene expression signatures of T cell dysfunction, T cell exclusion, myeloid-derived suppressor cell, cancer-associated fibroblast, and tumor associated macrophage M2 type [1]

## Conclusions

In conclusion, we present a TIDE web platform to infer gene functions in modulating tumor immunity and evaluate biomarkers to predict ICB clinical response. Our work underlines the value of data sharing of published trials and code sharing of published biomarkers. Notably, several published ICB clinical studies have not released their omics data or clinical data (Additional file 2: Table S2), and we hope their authors could release these data to bring invaluable resource to the whole research community. As the immunotherapy data becomes increasingly available, we foresee the TIDE web platform with increased value and benefit to the mechanism studies in cancer immunology and the biomarker discoveries in immune oncology.

## Supplementary information

Additional file 1: **Table S1.** New function modules and datasets in the TIDE web server.

Additional file 2: **Table S2.** Data availability of published ICB studies.

Additional file 3: **Table S3.** An example gene set related with immunotherapy response.

Additional file 4: **Figure S1.** The prediction performance of recently published biomarkers varies across data cohorts; and **Supplementary Methods**.

Additional file 5: **Table S4.** Genes with approved drugs.

Additional file 6: **Table S5.** Publications for public immunotherapy biomarkers.

## Data Availability

All the processed data can be accessed on http://tide.dfci.harvard.edu/. We collected ICB-naive cancer data sets with both patient survival durations and tumor gene expression profiles from the TCGA [2], METABRIC [3], and PRECOG [4] databases. Following the accession instruction described in published ICB studies (Additional file 2: Table S2), we downloaded ICB patients’ RNA-Seq raw sequencing data, clinical outcome information, and response outcome information from ICB studies (if available). The raw count table and meta-information of eight published CRISPR screens [5–8] were also obtained from the original studies. The list of genes with launched drugs, collected from the OASIS database [9], is available in Additional file 5: Table S4. The literature support of transcriptomic biomarkers is available in Additional file 6: Table S5.

## Abbreviations

CRISPR: Clustered regularly interspaced short palindromic repeats
ICB: Immune checkpoint blockade
TIDE: Tumor Immune Dysfunction and Evolution
