# Supplementary material for: Large-scale public data reuse to model immunotherapy response and resistance
Source: Genome Med. 2020 Feb 26;12:21. doi: 10.1186/s13073-020-0721-z (PMC7045518; doi:10.1186/s13073-020-0721-z)
Supplement: Supplementary file 1 — Table S1. New function modules and datasets in the TIDE web server. [file 13073_2020_721_MOESM1_ESM.docx]

| **Functions** |  | Original | New |
| --- | --- | --- | --- |
|  | Response prediction | - TIDE score - Dysfunction score - Exclusion score - CTL.flag - MDSC - CAF - TAM M2 | - TIDE score - Dysfunction score - Exclusion score - CTL.flag - MDSC - CAF - TAM M2 - MSI score - IFNG signature score - CD274 - CD8 - T clonality - B clonality |
|  | Regulator prioritization | NOT Available | Available |
|  | Biomarker evaluation | NOT Available | Available |
| **Datasets** | TCGA+PRECOG+METABRIC (n=33K) | Available | Available |
|  | CRISPR Dataset (studies = 8) | NOT Available | Available |
|  | Immunotherapy Dataset (n= 998) | NOT Available | Available |

**Table S1. New function modules and datasets in the TIDE web server.** All new components are highlighted in comparison to the previous version of TIDE web server since the initial publication of our manuscript [1].

**Reference**

1. Jiang P, Gu S, Pan D, Fu J, Sahu A, Hu X, Li Z, Traugh N, Bu X, Li B, et al: **Signatures of T cell dysfunction and exclusion predict cancer immunotherapy response.** *Nat Med* 2018, **24:**1550-1558.
