# Supplementary material for: Large-scale public data reuse to model immunotherapy response and resistance
Source: Genome Med. 2020 Feb 26;12:21. doi: 10.1186/s13073-020-0721-z (PMC7045518; doi:10.1186/s13073-020-0721-z)
Supplement: Supplementary file 2 — Table S2. Data availability of published ICB studies. [file 13073_2020_721_MOESM2_ESM.docx]

| **ICB Cohorts** | **Data Public Available** | | | **# of Patients (Not Available)** | **# of Patients (Included)** |
| --- | --- | --- | --- | --- | --- |
|  | **Transcriptome** | **Response Outcome** | **Survival Outcome** |  |  |
| Van Allen et al., Science 2015 [1] | Yes | Yes | Yes |  | 42 |
| Chen et al., Cancer Discov 2016 [2] | Yes | Yes | No |  | 33 |
| Hugo et al., Cell 2016 [3] | Yes | Yes | Yes |  | 25 |
| Lauss et al., Nat Commun 2017 [4] | Yes | Yes | Yes |  | 28 |
| Ayers et al., J Clin Invest 2017 [5] | No | No | No | 81 | \ |
| Prat et al., Cancer Res 2017 [6] | Yes | Yes | Yes |  | 33 |
| Nathanson et al., Cancer Immunol Res 2017 [7] | Yes | Yes | Yes |  | 24 |
| Riaz et al., Cell 2017 [8] | Yes | Yes | Yes |  | 51 |
| Miao et al., Science 2018 [9] | Yes | Yes | Yes |  | 33 |
| Mariathasan et al., Nature 2018 [10] | Yes | Yes | Yes |  | 348 |
| Kim et al., Nat Medicine 2018 [11] | Yes | Yes | No |  | 45 |
| McDermott et al., Nat Medicine 2018 [12] | Yes | Yes | No |  | 263 |
| Rodig et al., Sci Transl Med 2018 [13] | No | No | No | 181 | \ |
| Cristescu et al., Science 2018 [14] | No | No | No | 315 | \ |
| Gide et al., Cancer Cell 2019 [15] | Yes | Yes | Yes |  | 73 |

**Table S2. Data availability of published ICB studies.** All studies are annotated separately by the availability of transcriptomic data, response outcome, and survival outcome.

**Reference**

1. Van Allen EM, Miao D, Schilling B, Shukla SA, Blank C, Zimmer L, Sucker A, Hillen U, Foppen MHG, Goldinger SM, et al: **Genomic correlates of response to CTLA-4 blockade in metastatic melanoma.** *Science* 2015, **350:**207-211.

2. Chen PL, Roh W, Reuben A, Cooper ZA, Spencer CN, Prieto PA, Miller JP, Bassett RL, Gopalakrishnan V, Wani K, et al: **Analysis of Immune Signatures in Longitudinal Tumor Samples Yields Insight into Biomarkers of Response and Mechanisms of Resistance to Immune Checkpoint Blockade.** *Cancer Discov* 2016, **6:**827-837.

3. Hugo W, Zaretsky JM, Sun L, Song C, Moreno BH, Hu-Lieskovan S, Berent-Maoz B, Pang J, Chmielowski B, Cherry G, et al: **Genomic and Transcriptomic Features of Response to Anti-PD-1 Therapy in Metastatic Melanoma.** *Cell* 2016, **165:**35-44.

4. Lauss M, Donia M, Harbst K, Andersen R, Mitra S, Rosengren F, Salim M, Vallon-Christersson J, Torngren T, Kvist A, et al: **Mutational and putative neoantigen load predict clinical benefit of adoptive T cell therapy in melanoma.** *Nat Commun* 2017, **8:**1738.

5. Ayers M, Lunceford J, Nebozhyn M, Murphy E, Loboda A, Kaufman DR, Albright A, Cheng JD, Kang SP, Shankaran V, et al: **IFN-gamma-related mRNA profile predicts clinical response to PD-1 blockade.** *J Clin Invest* 2017, **127:**2930-2940.

6. Prat A, Navarro A, Pare L, Reguart N, Galvan P, Pascual T, Martinez A, Nuciforo P, Comerma L, Alos L, et al: **Immune-Related Gene Expression Profiling After PD-1 Blockade in Non-Small Cell Lung Carcinoma, Head and Neck Squamous Cell Carcinoma, and Melanoma.** *Cancer Res* 2017, **77:**3540-3550.

7. Nathanson T, Ahuja A, Rubinsteyn A, Aksoy BA, Hellmann MD, Miao D, Van Allen E, Merghoub T, Wolchok JD, Snyder A, Hammerbacher J: **Somatic Mutations and Neoepitope Homology in Melanomas Treated with CTLA-4 Blockade.** *Cancer Immunol Res* 2017, **5:**84-91.

8. Riaz N, Havel JJ, Makarov V, Desrichard A, Urba WJ, Sims JS, Hodi FS, Martin-Algarra S, Mandal R, Sharfman WH, et al: **Tumor and Microenvironment Evolution during Immunotherapy with Nivolumab.** *Cell* 2017, **171:**934-949 e915.

9. Miao D, Margolis CA, Gao W, Voss MH, Li W, Martini DJ, Norton C, Bosse D, Wankowicz SM, Cullen D, et al: **Genomic correlates of response to immune checkpoint therapies in clear cell renal cell carcinoma.** *Science* 2018, **359:**801-806.

10. Mariathasan S, Turley SJ, Nickles D, Castiglioni A, Yuen K, Wang Y, Kadel Iii EE, Koeppen H, Astarita JL, Cubas R, et al: **TGFbeta attenuates tumour response to PD-L1 blockade by contributing to exclusion of T cells.** *Nature* 2018, **554:**544-548.

11. Kim ST, Cristescu R, Bass AJ, Kim KM, Odegaard JI, Kim K, Liu XQ, Sher X, Jung H, Lee M, et al: **Comprehensive molecular characterization of clinical responses to PD-1 inhibition in metastatic gastric cancer.** *Nat Med* 2018, **24:**1449-1458.

12. McDermott DF, Huseni MA, Atkins MB, Motzer RJ, Rini BI, Escudier B, Fong L, Joseph RW, Pal SK, Reeves JA, et al: **Clinical activity and molecular correlates of response to atezolizumab alone or in combination with bevacizumab versus sunitinib in renal cell carcinoma.** *Nat Med* 2018, **24:**749-757.

13. Rodig SJ, Gusenleitner D, Jackson DG, Gjini E, Giobbie-Hurder A, Jin C, Chang H, Lovitch SB, Horak C, Weber JS, et al: **MHC proteins confer differential sensitivity to CTLA-4 and PD-1 blockade in untreated metastatic melanoma.** *Sci Transl Med* 2018, **10**.

14. Cristescu R, Mogg R, Ayers M, Albright A, Murphy E, Yearley J, Sher X, Liu XQ, Lu H, Nebozhyn M, et al: **Pan-tumor genomic biomarkers for PD-1 checkpoint blockade-based immunotherapy.** *Science* 2018, **362**.

15. Gide TN, Quek C, Menzies AM, Tasker AT, Shang P, Holst J, Madore J, Lim SY, Velickovic R, Wongchenko M, et al: **Distinct Immune Cell Populations Define Response to Anti-PD-1 Monotherapy and Anti-PD-1/Anti-CTLA-4 Combined Therapy.** *Cancer Cell* 2019, **35:**238-255 e236.
