# Supplementary material for: Large-scale public data reuse to model immunotherapy response and resistance
Source: Genome Med. 2020 Feb 26;12:21. doi: 10.1186/s13073-020-0721-z (PMC7045518; doi:10.1186/s13073-020-0721-z)
Supplement: Supplementary file 3 — Table S3. An example gene set related with immunotherapy response. [file 13073_2020_721_MOESM3_ESM.docx]

| **Gene** | **weight** | **Evidence Reference** |
| --- | --- | --- |
| *SERPINB9* | -1.0 | [1] |
| *TGFB1* | -1.0 | [2] |
| *PDL1* | 1.0 | [3] |
| *FAP* | -1.0 | [4] |
| *VEGFA* | -1.0 | [5] |
| *IFNG* | 1.0 | [6] |
| *ANGPT2* | -1.0 | [7] |

**Table S3. An example gene set related with immunotherapy response.** All members in the set have literature supports for their values in predicting immunotherapy outcome.

**Reference**

1. Jiang P, Gu S, Pan D, Fu J, Sahu A, Hu X, Li Z, Traugh N, Bu X, Li B, et al: **Signatures of T cell dysfunction and exclusion predict cancer immunotherapy response.** *Nat Med* 2018, **24:**1550-1558.

2. Mariathasan S, Turley SJ, Nickles D, Castiglioni A, Yuen K, Wang Y, Kadel Iii EE, Koeppen H, Astarita JL, Cubas R, et al: **TGFbeta attenuates tumour response to PD-L1 blockade by contributing to exclusion of T cells.** *Nature* 2018, **554:**544-548.

3. Nishino M, Ramaiya NH, Hatabu H, Hodi FS: **Monitoring immune-checkpoint blockade: response evaluation and biomarker development.** *Nat Rev Clin Oncol* 2017.

4. Feig C, Jones JO, Kraman M, Wells RJ, Deonarine A, Chan DS, Connell CM, Roberts EW, Zhao Q, Caballero OL, et al: **Targeting CXCL12 from FAP-expressing carcinoma-associated fibroblasts synergizes with anti-PD-L1 immunotherapy in pancreatic cancer.** *Proc Natl Acad Sci U S A* 2013, **110:**20212-20217.

5. Voron T, Colussi O, Marcheteau E, Pernot S, Nizard M, Pointet AL, Latreche S, Bergaya S, Benhamouda N, Tanchot C, et al: **VEGF-A modulates expression of inhibitory checkpoints on CD8+ T cells in tumors.** *J Exp Med* 2015, **212:**139-148.

6. Ayers M, Lunceford J, Nebozhyn M, Murphy E, Loboda A, Kaufman DR, Albright A, Cheng JD, Kang SP, Shankaran V, et al: **IFN-gamma-related mRNA profile predicts clinical response to PD-1 blockade.** *J Clin Invest* 2017, **127:**2930-2940.

7. Schmittnaegel M, Rigamonti N, Kadioglu E, Cassara A, Wyser Rmili C, Kiialainen A, Kienast Y, Mueller HJ, Ooi CH, Laoui D, De Palma M: **Dual angiopoietin-2 and VEGFA inhibition elicits antitumor immunity that is enhanced by PD-1 checkpoint blockade.** *Sci Transl Med* 2017, **9**.
