# Supplementary material for: Large-scale public data reuse to model immunotherapy response and resistance
Source: Genome Med. 2020 Feb 26;12:21. doi: 10.1186/s13073-020-0721-z (PMC7045518; doi:10.1186/s13073-020-0721-z)
Supplement: Supplementary file 4 — Figure S1. The prediction performance of recently published biomarkers varies across data cohorts; and Supplementary Methods. [file 13073_2020_721_MOESM4_ESM.docx]

**Fig S1. The prediction performance of recently published biomarkers varies across data cohorts.**

1. Prediction performance of recently published biomarkers [1, 2] was evaluated by the biomarker evaluation module in the TIDE web platform, using a gene set approach.
2. The prediction performance of immune resistance score [2] calculated following the authors’ original scripts across all melanoma cohorts.

**Supplementary Methods**

**Data processing**

For each RNA-seq dataset, the transcriptomic profile was log2(1+TPM) transformed. We standardized the log scale transcriptome data across patients by quantile-normalization, and further normalized the expression values of each gene by subtracting the average among all samples. For each CRISPR Screen dataset, on the level of each guide RNA, we computed log2 fold change (logFC) of counts-per-million reads between treatment and control conditions. Then, the median logFC among all guides was reported for each gene.

**Biomarkers calculation**

The predicted values of gene expression biomarkers (CD274, CD8, IFNG) were the average expression values among all members (Additional file 6: Table S5). The TIDE prediction value is computed following the procedure in the original publication [3]. Of all collected ICB trails, seven provide mutation profile. The predicted value of tumor mutation burden (TMB) was the total amount of non-synonymous mutation. We applied the MiXCR algorithm on RNA-Seq sequencing data to obtain T and B cell receptor CDR3 sequences for every patient [4]. The predicted values of T cell clonality and B cell clonality were calculated by $1-\sum_{i=1}^{N} p_{i} \frac{log\frac{1}{p_{i}}}{\mathrm{logN}}$ ($p_{i}$: the frequency of each receptor sequence). In TCGA dataset, there are nine cancer types with microsatellite instability (MSI) information but are only three (STAD, COAD, and UCEC) with compatible numbers (the proportion > 30%) of positive and negative labels. Thus, we trained ridge regression models on gene expression profile to predict MSI on those three cancer types separately. The best model for every cancer type was chosen by the leave-one-out cross-validation method. Then, cancer-specific models were validated on tumors from other two types. The model trained on STAD has the highest AUC score on the validation step. We implemented model trained on STAD to evaluate MSI status on the website.

**Reference**

1. Shukla SA, Bachireddy P, Schilling B, Galonska C, Zhan Q, Bango C, Langer R, Lee PC, Gusenleitner D, Keskin DB, et al: **Cancer-Germline Antigen Expression Discriminates Clinical Outcome to CTLA-4 Blockade.** *Cell* 2018, **173:**624-633 e628.

2. Jerby-Arnon L, Shah P, Cuoco MS, Rodman C, Su MJ, Melms JC, Leeson R, Kanodia A, Mei S, Lin JR, et al: **A Cancer Cell Program Promotes T Cell Exclusion and Resistance to Checkpoint Blockade.** *Cell* 2018, **175:**984-997 e924.

3. Jiang P, Gu S, Pan D, Fu J, Sahu A, Hu X, Li Z, Traugh N, Bu X, Li B, et al: **Signatures of T cell dysfunction and exclusion predict cancer immunotherapy response.** *Nat Med* 2018, **24:**1550-1558.

4. Bolotin DA, Poslavsky S, Davydov AN, Frenkel FE, Fanchi L, Zolotareva OI, Hemmers S, Putintseva EV, Obraztsova AS, Shugay M, et al: **Antigen receptor repertoire profiling from RNA-seq data.** *Nat Biotechnol* 2017, **35:**908-911.
