# Supplementary material for: Large-scale public data reuse to model immunotherapy response and resistance
Source: Genome Med. 2020 Feb 26;12:21. doi: 10.1186/s13073-020-0721-z (PMC7045518; doi:10.1186/s13073-020-0721-z)
Supplement: Supplementary file 5 — Table S4. Genes with approved drugs. [file 13073_2020_721_MOESM5_ESM.docx]

**Table S4. Genes with approved drugs.** The list of genes with launched drugs is derived from the OASIS database *[1]*.

**Reference**

1. Fernandez-Banet J, Esposito A, Coffin S, Horvath IB, Estrella H, Schefzick S, Deng S, Wang K, K AC, Ding Y, et al: **OASIS: web-based platform for exploring cancer multi-omics data.** *Nat Methods* 2016, **13:**9-10.
