# Supplementary material for: Large-scale public data reuse to model immunotherapy response and resistance
Source: Genome Med. 2020 Feb 26;12:21. doi: 10.1186/s13073-020-0721-z (PMC7045518; doi:10.1186/s13073-020-0721-z)
Supplement: Supplementary file 6 — Table S5. Publications for public immunotherapy biomarkers. [file 13073_2020_721_MOESM6_ESM.docx]

| **Biomarkers** | **Publications** |
| --- | --- |
| CD274 | [1] |
| CD8 | Average expression of CD8A + CD8B [2] |
| IFNG | [3] |
| TIDE | [4] |

**Table S5. Publications for public immunotherapy biomarkers.**

**Reference**

1. Nishino M, Ramaiya NH, Hatabu H, Hodi FS: **Monitoring immune-checkpoint blockade: response evaluation and biomarker development.** *Nat Rev Clin Oncol* 2017.

2. Chen PL, Roh W, Reuben A, Cooper ZA, Spencer CN, Prieto PA, Miller JP, Bassett RL, Gopalakrishnan V, Wani K, et al: **Analysis of Immune Signatures in Longitudinal Tumor Samples Yields Insight into Biomarkers of Response and Mechanisms of Resistance to Immune Checkpoint Blockade.** *Cancer Discov* 2016, **6:**827-837.

3. Ayers M, Lunceford J, Nebozhyn M, Murphy E, Loboda A, Kaufman DR, Albright A, Cheng JD, Kang SP, Shankaran V, et al: **IFN-gamma-related mRNA profile predicts clinical response to PD-1 blockade.** *J Clin Invest* 2017, **127:**2930-2940.

4. Jiang P, Gu S, Pan D, Fu J, Sahu A, Hu X, Li Z, Traugh N, Bu X, Li B, et al: **Signatures of T cell dysfunction and exclusion predict cancer immunotherapy response.** *Nat Med* 2018, **24:**1550-1558.
